# Fast and Robust Characterization of Time-Heterogeneous Sequence Evolutionary Processes Using Substitution Mapping

**DOI:** 10.1371/journal.pone.0033852

**Published:** 2012-03-27

**Authors:** Jonathan Romiguier, Emeric Figuet, Nicolas Galtier, Emmanuel J. P. Douzery, Bastien Boussau, Julien Y. Dutheil, Vincent Ranwez

**Affiliations:** 1 Institut des Sciences de l'Evolution de Montpellier, CNRS-Université Montpellier 2, Montpellier, France; 2 Laboratoire de Biométrie et Biologie Evolutive, CNRS-Université Lyon 1, Villeurbanne, France; 3 Unité Mixte de Recherche Amélioration génétique et adaptation des plantes méditerranéennes et tropicales, Montpellier SupAgro, Montpellier, France; 4 Department of Integrative Biology, University of California, Berkeley, California, United States of America; University of Wyoming, United States of America

## Abstract

Genes and genomes do not evolve similarly in all branches of the tree of life. Detecting and characterizing the heterogeneity in time, and between lineages, of the nucleotide (or amino acid) substitution process is an important goal of current molecular evolutionary research. This task is typically achieved through the use of non-homogeneous models of sequence evolution, which being highly parametrized and computationally-demanding are not appropriate for large-scale analyses. Here we investigate an alternative methodological option based on probabilistic substitution mapping. The idea is to first reconstruct the substitutional history of each site of an alignment under a homogeneous model of sequence evolution, then to characterize variations in the substitution process across lineages based on substitution counts. Using simulated and published datasets, we demonstrate that probabilistic substitution mapping is robust in that it typically provides accurate reconstruction of sequence ancestry even when the true process is heterogeneous, but a homogeneous model is adopted. Consequently, we show that the new approach is essentially as efficient as and extremely faster than (up to 25 000 times) existing methods, thus paving the way for a systematic survey of substitution process heterogeneity across genes and lineages.

## Introduction

Mapping the history of nucleotide or amino-acid changes onto the evolutionary history of a gene, as depicted by a phylogenetic tree, is of central interest to researchers in molecular evolution. This procedure, called mutation or substitution mapping, is useful for characterizing the molecular evolutionary processes of DNA and protein sequences, and their variations across sites and lineages. Substitution mapping has been successfully applied to study various aspects of molecular evolution, including coevolution [1], [2], selective constraints in proteins [3], deviations from the molecular clock hypothesis [4], and changes in selective regimes [5]. Beyond this, substitution mapping has also enabled the implementation of a number of models that were otherwise intractable [6],[7].

Over the past 10 years, several inference methods have been developed to achieve substitution mapping. Formally, the problem is to identify, for every site in a sequence alignment, the kinds of character changes that occurred, and their location in the underlying phylogeny. So a substitution mapping method would take an alignment and a tree as input and return, as output, an estimate of the number/nature of substitutions that have occurred, for each site of the alignment and each branch of the tree. The “naive” substitution mapping procedure [8] involves first reconstructing all ancestral sequences at each node of the phylogenetic tree. Secondly, for each site, one substitution is mapped on a branch when two different states are observed for this site at the two extremities of the branch. The main drawback of such an approach is that it overlooks the uncertainty of the ancestral sequence inference.

Two improved mapping methods have been proposed: Bayesian Mutational Mapping (BMM, [9]) and Probabilistic Substitution Mapping (PSM, [1],[10]). They both use Markov chains to model the substitution process and account for the uncertainty in the ancestral states [11], [12]. BMM is a procedure that generates a substitution scenario compatible with the data, together with its associated likelihood. This procedure was not designed to produce human-readable substitution maps, but rather to integrate a statistic of interest over the set of possible substitution maps. Because it is a sampling procedure, BMM is fairly computer-expensive, although some more stable or efficient samplers have been proposed lately [13], [14], [15]. PSM is an analytical procedure, which computes the probability distribution of the number of substitutions that occurred at each site of the alignment and each branch of the phylogenetic tree. Dutheil et al 2005 [1] report how to compute the mean number of total substitutions per branch and site, but it is also possible to compute higher-order moments of the distribution, or distinguish between different types of substitutions ([14],[15] and the present study). PSM is a maximum likelihood solution of BMM for some particular statistics (the mean of the branch and site-specific distributions of the expected number of substitutions in the case of Dutheil et al 2005 [1]) and is therefore quite fast to compute for a given tree and substitution model, which is a significant advantage with respect to the increasing amount of molecular data provided by high-throughput sequencing. In addition, the relative simplicity and computer efficiency of substitution mapping procedures have promoted them for use in several analyses (e.g. [16]). They have been shown to facilitate parameter estimation of complex models when used within expectation-maximization procedures [17]. Adequate statistics based on substitution maps could therefore serve as straight-forward descriptors of molecular evolution that can be used as proxies for more complex ones.

One of the major advantages of substitution mapping is its power to detect and characterize time-heterogeneous processes, i.e. processes that vary across branches of the tree. Such variations, when identified, can be linked to variations in selective pressure (e.g. [18]) and mutation/fixation biases (e.g. [19]), or linked to macroscopic features of species such as effective population size (e.g. [20], [21]), ecological preferences [22] or life-history traits [23]. To detect heterogeneous processes, explicit models of non-homogeneous sequence evolution have been implemented in the maximum-likelihood or Bayesian frameworks [22], [24], [25], [26]. However, these parameter-rich models could lead to over-parametrization issues and are computationally demanding, so their usage is limited to relatively small subsets of the large amounts of currently available sequence data. Being fast and flexible, substitution mapping may potentially offer the opportunity to detect heterogeneous processes without fitting parameter-rich heterogeneous models. One possibility would be to map substitutions under a simple, fast, time-homogeneous model of sequence evolution, and then rely on the inferred changes to assess the heterogeneity of the evolutionary process, at low computational cost.

This, however, raises concern as to the ability of substitution mapping procedures to infer characteristics of the data which are not explicitly hard-coded in the model used for the mapping. This study is the first attempt to assess the extent to which substitution mapping is robust with respect to time-heterogeneous model choice. Using simulations under realistic non-homogeneous models of substitutions, both at the nucleotide and codon level, we demonstrate that probabilistic substitution mapping is robust to the *a priori* choice of substitution model. We show that even a homogeneous model with roughly approximate branch lengths captures most of the signal in the data, and allows to very efficiently infer complex aspects of the real process, including non-homogeneity. A dataset of 139 mammalian mitochondrial genomes and 243 ribosomal DNA sequences (18 S) from vertebrates is then used to illustrate the scalability of this method. Finally, we tested the method as a substitute to the famous *codeml* software from the PAML package [27] for large database analysis. Based on 993 vertebrate gene families from the *Selectome* database [28], we show that substitution mapping is a faster and better way to describe variations in the substitution process across each branch of a tree.

## Results and Discussion

### Analyses of simulations at the codon level

To test the robustness of substitution mapping, we propose to evaluate its ability to infer the dN/dS (non-synonymous/synonymous substitutions) ratio under various conditions. For this, we simulated 50 alignments of 1000 sites under a non-homogeneous YN98 codon model [25]. This model assumes a distinct omega value (dN/dS) for each branch. From a 33-leaf tree, we obtained 33 simulated sequences and 63 dN/dS ratios for each branch of the phylogeny. The tree topology was taken from reference [23]. Branch lengths were estimated from a real data set of 987 orthologous genes in 33 mammals obtained from the OrthoMam database [29] (Figure S1).

Substitution mapping was then used on these data. Directly inferred from sequences, this mapping provides synonymous and non-synonymous substitution counts, and allows deduction of a dN/dS ratio per branch. Since it is based on homogeneous models, this dN/dS estimation strategy does not require long optimization of multiple omega parameters. To test its model tolerance, substitution mapping was performed under different substitution models, i) a non-homogeneous YN98 model, the same used in simulations, ii) a homogeneous YN98 model, with a single omega value shared by all branches, iii) a Jukes Cantor model [30], where all substitution rates are fixed and equal.

Branch lengths and parameter values were re-estimated under these three models before substitution mapping computations. Because these re-estimations represent the largest percentage of the computation time, it is interesting to assess their real impact on substitution mapping. We thus added a fourth substitution mapping condition to test the robustness of substitution mapping with respect to branch length parameters. For this purpose, we performed substitution mapping under a homogeneous YN98 model with fixed branch lengths, randomly distorted values (in a range of+/−25% of their original value).

**Figure 1:**
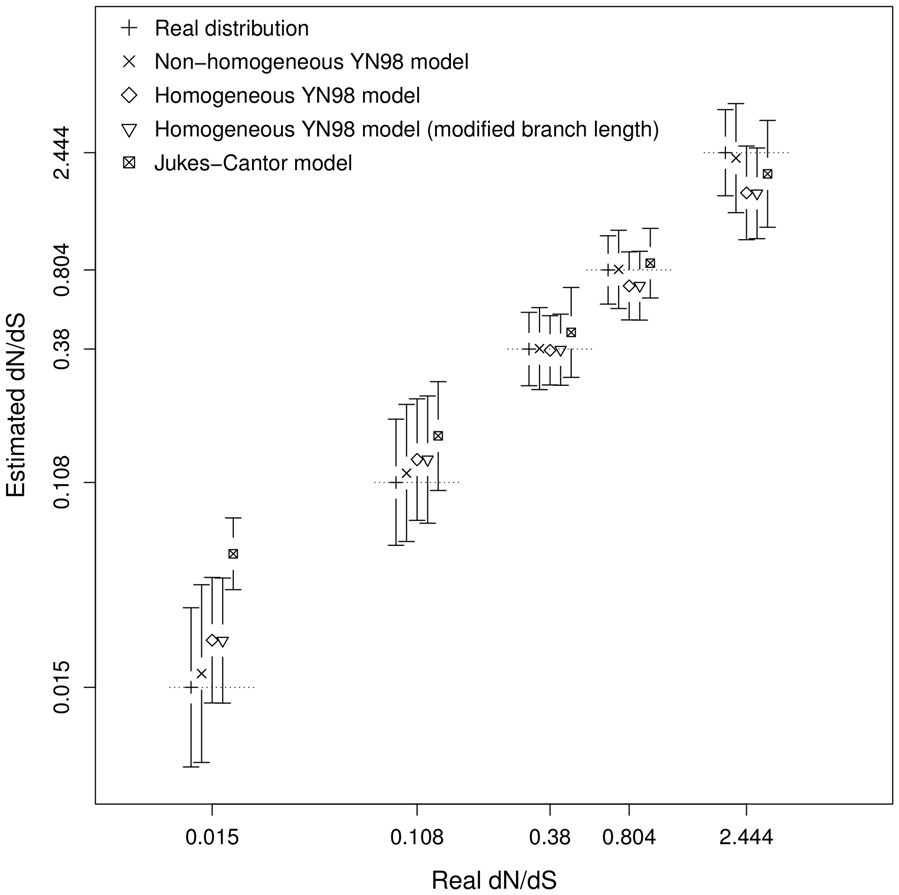
Estimated dN/dS under various substitution mapping conditions compared with real dN/dS from simulations. Axes are in log scale. Each point represents the median of one of the five classes. Bars represent the median absolute deviation of their corresponding class. The real distribution is plotted to give an idea of the initial variability of values to estimate.

The simulation results are summarized in figure 1, and allow comparison of the dN/dS ratio deduced from substitution mapping with the real one resulting from the sequence simulation. Overall, the dN/dS ratio is well deduced by substitution mapping and estimated distributions are in the range of the corresponding simulated ones. Using a homogeneous or non-homogeneous model for the mapping seems to have very little effect. A single omega value shared by all branches already provides a correct estimation of the real molecular evolution process where the omega value varies across branches. Indeed, even the Jukes-Cantor model seems to provide reliable dN/dS estimation, despite the fact that it does not distinguish synonymous from non-synonymous substitution rates. Note, however, that it performs slightly worse than other models for very low omega values (<0.02). Since the Jukes-Cantor model assumes equal non-synonymous and synonymous substitution parameters, it tends to over-estimate dN when omega is ≪1.

Another major result of this analysis is the very strong similarity between inferences, conducted with and without distorted branch lengths. For a homogeneous model, using correct branch length values does not improve the dN/dS inference by substitution mapping. This result promises very short computation time for substitution mapping, since a rough estimation of branch lengths is sufficient to obtain a reliable inference of molecular evolution processes.

**Figure 2:**
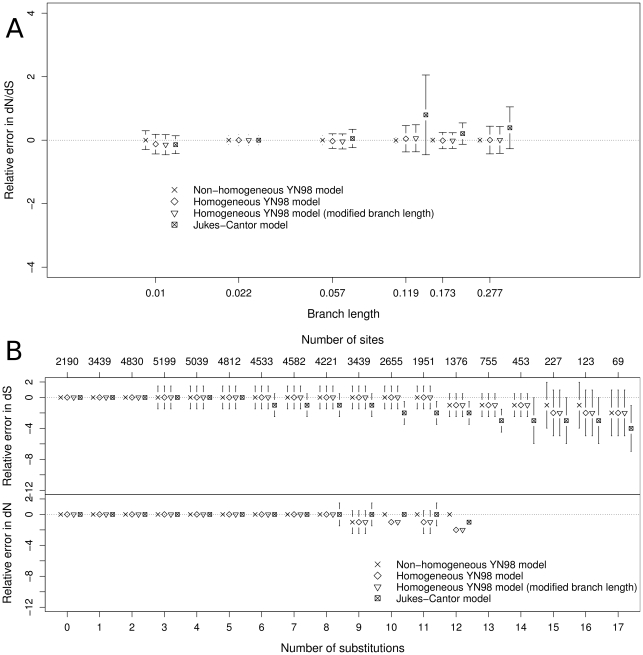
Relative error in dN/dS estimation by branch and by site. Panel A is the relative error variation in dN/dS estimation of a branch according to its branch length value. The branch length axis is in log scale. Each point represents the median of one of the six classes, bars represent the median absolute deviation of their corresponding class. Panel B is the relative error in dN and dS of a site according to its synonymous substitution number (top plot) and non-synonymous substitution number (bottom plot) in all the branches of the tree. Each point represents the median of one of the 17 classes (for readability, we do not show the 5 last classes that concern only 0.2% of our sites), bars represent the median absolute deviation of their corresponding class. The top axis gives the number of sites concerned by the corresponding number of synonymous substitutions.

Figure 2 shows the influence of the branch length (fig. 2A) and substitution rate (fig. 2B) on dN and dS estimation accuracy. Our substitution mapping estimations are evaluated according to their relative error in dN/dS, defined as follows:
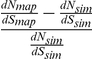
where 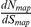 is the dN/dS ratio obtained from mapping substitutions, and 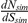 is the true dN/dS observed during simulations which generate sequences.

As we can see in figure 2A, branch lengths seem to have no major effect with YN98 models (non-homogeneous or homogeneous), but the Jukes-Cantor model tends to over-estimate dN/dS, and the relative error of this estimation increases with the branch length.

This effect could be explained by the occurrence of multiple substitutions. When a site presents a single substitution on a particular branch, the mapping method easily discriminates between a synonymous and a non-synonymous substitution, whatever the model used. On the other hand, in case of multiple substitutions, their distribution in non-synonymous or synonymous categories is mostly determined by the model, and forced to occur at an equal rate with the Jukes-Cantor model. As expected, this bias is stronger for long branches (figure 2A) and fast evolving sites (figure 2B). The Jukes-Cantor model clearly underestimates synonymous substitutions at sites where multiple substitutions take place. This effect also occurs with YN98 models, but affects sites much less. On the other hand, the Jukes-Cantor model does not seem to underestimate non-synonymous substitutions, even when there are 9 or more substitutions. This could be explained by the fact that the Jukes-Cantor model always overestimates dN because of its lack of omega value which implies equal synonymous and non-synonymous substitution probabilities. Note that a large majority of sites are subjected to fewer than 10 substitutions on the whole tree, a threshold where substitution mapping with a homogeneous YN98 model gives results similar to those obtained with a non-homogeneous model.

Note that there is no correlation between branch depth and relative error (Pearson's R^2^ = 0.0002, p-value = 0.45 for homogeneous T92 model, similar results are obtained with other models).

In conclusion, the simulation results show very similar performances with respect to non-homogeneous and homogeneous substitution mapping for estimating the dN/dS ratio. With a single omega value, the homogeneous YN98 model captures the evolutionary history of sequences that evolved under distinct substitution processes. In spite of its simplicity, the Jukes-Cantor model shows comparable performances, except in the presence of long branches, where multiple substitutions can lead it to over-estimate dN/dS ratio (by underestimating dS and overestimating dN). For highly divergent datasets, the homogeneous YN98 model therefore generates more accurate estimations, while still saving the time cost of a full-optimized non-homogeneous model.

### Analysis of simulations at the nucleotide level

Simulation analyses were performed on GC-content patterns using a simulation protocol similar to that used for dN/dS. We simulated 50 000 sites under a non-homogeneous model [24], which assumes a different GC-equilibrium (theta value) per branch. We used the same topology (33 leaf tree) as that used for codon simulations.

We then tested the ability of substitution mapping to infer the proportion of (A or T) to (G or C) substitution, under different conditions: i) a non-homogeneous model, ii) a homogeneous T92 model [31], iii) a homogeneous T92 model with a distorted branch length tree, iv) a homogeneous Jukes-Cantor model. These results (presented here as supplementary material, Figure S2 and Figure S3) led to conclusions similar to those of previous codon simulations: substitution mapping with a homogeneous model of sequence evolution is sufficient to recover variations in AT to GC and GC to AT substitutions throughout the phylogeny.

### dN/dS estimation on data from mammalian mitochondria

The ability of substitution mapping to estimate heterogeneous substitution processes from a simple homogeneous model provides an opportunity to achieve a great speed boost for dN/dS inference.

For several reasons, this molecular evolutionary aspect has attracted considerable attention over the last years.

Because dN/dS is expected to be equal to 1 in case of neutral evolution, this ratio can give some clues about natural selection pressure on molecular evolution. Such traces of positive selection have been detected, revealing genetic differentiation to herbivory [27] or genes under selection in the human genome [32]. Furthermore, dN/dS provides a way to detect life history trait imprints on molecular sequences. According to the nearly neutral theory of molecular evolution [33], most non-synonymous substitutions are slightly deleterious. Because of stronger genetic drift, species with a small population size (*Ne*) are more prone to accumulate these slightly deleterious mutations. This less effective purifying selection leads to a higher dN/dS ratio in large mammal species which exhibit small *Ne* in comparison to small species [21]. Similar effects have been found between lineages with asexual or sexual reproductive modes [20], [34], [35].

These links between molecular evolution, natural selection and life history traits are used to an increasing extent in analyses with the dN/dS ratio, typically conducted with the highly popular *PAML* [27] or *HyPhy* [36] packages. Such programs infer a dN/dS ratio through non-homogeneous models, often with one-per-branch omega values, and can conduct likelihood comparison tests for statistical detection of positive selection. However, these procedures need to fit multiple parameters, which is not only computationally costly, but can also lead to poor estimations due to over-parameterization.

Our simulations show that substitution mapping using simple models with a single parameter is a relevant alternative. Substitution mapping provides one dN/dS ratio per branch without requiring complex multiple parameter optimizations, therefore providing the fastest and easiest way to perform dN/dS analyses. Furthermore, as substitution mapping is very robust to branch length errors, extra computation time can be saved using rough branch length optimization.

We used data similar to that used by Popadin et al [21] to further test this approach. From 139 mammalian mitochondrial genomes, they found positive correlations between dN/dS ratios (using *codeml*) and body mass, as expected through theoretical population size effects on purifying selection. Because estimating a dN/dS per branch was not feasible (due to computation time limitation), they conducted their analysis on 11 monophyletic subtrees. To assess the efficiency and the speed of our substitution mapping method, we used a similar dataset with more species (165), and estimated dN/dS ratios on the whole 165-tip tree.

**Figure 3:**
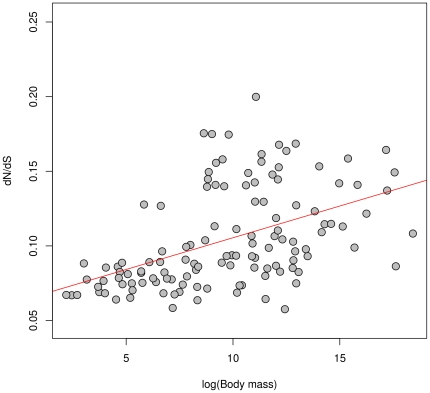
dN/dS ratio of terminal branches of 139 mammals according to their body mass.

Branch lengths and the single omega parameter value were estimated under an YN98 homogeneous model in BppML [37]. Since the simulations indicated a strong robustness to branch length errors, we estimated them with a low precision (optimization stopped when the improvements in log-likelihood were under 1 log likelihood point). We then used MapNH (software available at http://biopp.univ-montp2.fr/forge/testnh), to obtain dN and dS counts. The results of this analysis are shown in figure 3, where the estimated dN/dS of each terminal branch is compared to the body mass of the corresponding leaf. A strong significant correlation was found between these two variables (p<0.0001, Kendal's tau = 0.35) which is in agreement with the conclusions of Popadin et al and theoretical predictions. The total computation time of our substitution mapping procedure (including MapNH and prior estimation of parameters by BppML) was 1 h 34 min on a desktop computer (Intel Xeon 2.27Ghz CPU) whereas, by comparison, fitting an equivalent non-homogeneous model with the PAML package required 38 days.

Substitution mapping thus seems to be a reliable tool to perform dN/dS ratio analyses, while being remarkably faster than classical approaches (more than 60 times). Moreover, as mentioned in the introduction, substitution mapping is theoretically not limited to dN/dS and can explore all types of substitution process heterogeneity, as illustrated in the following section where mapping is used to explore GC heterogeneity.

### GC equilibrium estimation on data from vertebrate 18 S ribosomal RNA

Here, we tested the reliability of substitution mapping at the nucleotide level using a benchmark dataset with a documented variation in GC-content caused by biased gene conversion [38]. To evaluate the impact of biased gene conversion [39] on 18 S ribosomal RNA, Escobar et al [38] estimated the GC equilibrium of 243 vertebrate species through a non-homogeneous model. We used the alignments and tree provided with the article to conduct a mapping analysis (BppML parameter estimations followed by MapNH substitution mapping). We obtained (A or T) to (G or C) and (G or C) to (A or T) substitution counts for all branches of the tree, and computed a rough approximation of GC equilibrium defined by 
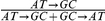
 where AT→GC and GC→AT are the substitution counts inferred by substitution mapping for each substitution type.

**Figure 4:**
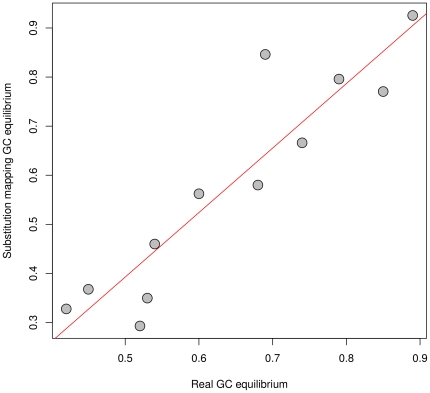
Estimation of equilibrium GC obtained by substitution mapping compared to equilibrium GC obtained with a non-homogeneous model (one GC equilibrium parameter per branch, values obtained from Escobar et al 2011).

We compared these substitution mapping estimations to GC-equilibrium values of the internal branches shown by Escobar et al in their figure 2. Because of the lack of statistical power for small branches (too few substitution events to compute a reliable GC-equilibrium), we excluded all branches with fewer than 10 substitutions, and report a significant correlation (Pearson's R^2^ = 0.85, p-value<0.0001) (figure 4). The total computation time was 44 s.

This comparison confirms that a simple homogeneous model used with substitution mapping can capture heterogeneous molecular evolution processes as well as massive time-consuming heterogeneous models. In this case, optimization through a non-homogeneous model takes more than 13 days, i.e. is more than 25 000 time slower than our substitution mapping approach. This huge gain of time, a lot better than for our codon case study, is probably due to various differences between the two datasets, particularly the number of species (here 243 compared to 165, i.e. more parameters per branch to estimate for the non-homogeneous model).

### Substitution mapping for scalable genomic-scale selection analyses

As reviewed before, dN/dS computations and searches for traces of selection in substitution patterns are popular analyses used in recently published studies [20], [21], [32], [34], [35]. Consequently, several databases include such analyses on a large amount of data: Human PAML browser [40], The Adaptative Evolution Database [41], or Selectome [28]. All of these databases use *codeml* from the PAML package (Yang 1998). Because of the recent increase in available high throughput sequencing data, it will be increasingly difficult to quickly update such databases. Even with future increases in computer performance, it seems nearly impossible to imagine that one-per-branch non-homogeneous models could perform whole dN/dS analyses, e.g. in recent projects like 10 000 vertebrate genomes [42]. Despite the lack of likelihood comparison tests to detect accurately positive selection, fast and robust substitution mapping provides a scalable alternative to *codeml* for massive dN/dS analyses.

As a proof-of-concept, we used 993 gene alignments from the *Selectome* database, and compared dN/dS computations from *codeml* and substitution mapping. With a one-per-branch dN/dS parameter value, the CPU computation time of *codeml* was approximately 1 month (740 h). The same task took 10.72 h using substitution mapping (*BppML* and *MapNH*).

**Figure 5:**
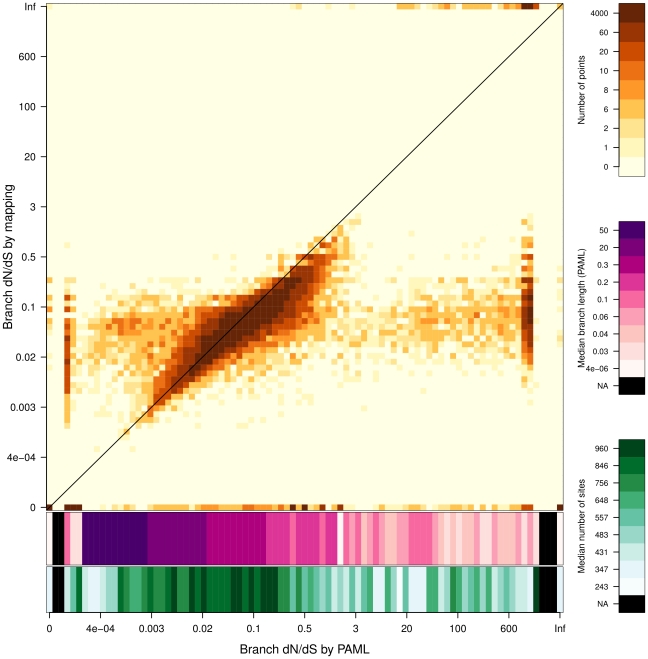
Estimation of dN/dS values by substitution mapping compared with dN/dS values estimated by PAML. Axes are in log scale. The orange color gradient represents the density of points. The two scales in purple and green show the median branch length (estimated by PAML) and the median number of sites for corresponding dN/dS values estimated by PAML. The 0 value includes estimations where dN is equal to 0 and estimations where dN and dS are both equal to 0 (0/0).

Figure 5 summarizes the PAML/mapping comparison for 30 643 dN/dS estimations (one per branch for 993 genes). dN/dS PAML estimations ranged from 0.003 to 3, and the heatmap highlights a high majority of correlated points. As seen before, this indicates that the two methods generated very similar results: 83.2% of our 30 643 dN/dS estimations are highly similar (less than 0.1 difference). Nevertheless, there is clearly a substantial number of outliers on both sides of this correlation line, revealing incongruent dN/dS values.

On the right of the plot, outliers are for a mainly due to extreme dN/dS values estimated by PAML, from 3 to over-represented aberrant dN/dS values near 1000 (2665 points). These erroneous values estimated by PAML are due to branches with very few substitution events, as illustrated by the two colored scales at the bottom, which indicate corresponding branch length values and the number of sites in the alignments. Under the same conditions, substitution mapping estimations were almost never above 3 (excepted for 310 infinite values, i.e. 0 synonymous substitutions detected), which makes more sense from a biological standpoint.

Outliers on the left of the plot correspond to very low dN/dS estimations by PAML (<0.002). As illustrated by the branch length color scale, these outliers are mainly due to aberrant branch length estimations obtained by PAML, with a median value ranging from 20 to 50. These extreme branch length values probably lead to overestimation of dS, and produce abnormally low dN/dS values for PAML. For those branches, substitution mapping probably benefits from its robustness to branch length estimation errors, and from the fact that those estimations are achieved through a more constrained homogeneous model. Whereas there are 693 branch length values above 10 with PAML, there are only 31 with BppML. This difference is probably caused by over-parametrization in the non-homogeneous PAML model, thus illustrating the technical difficulties that may arise when estimating numerous branch length values plus several substitution parameters simultaneously.

Outliers produced by substitution mapping are easily identifiable and correspond to 0 and infinite values, respectively, when there is 0 non-synonymous and 0 synonymous substitutions detected. They represent only 1.5% of points for plausible dN/dS values estimated by PAML (between 0.003 and 10), whereas PAML outliers (values lower than 0.003 and higher than 10 for plausible mapping values) represent 9% of points.

For our 30,643 estimations, 384 are greater than 1 with substitution mapping (74 without infinite values), and only 10 of these instances of positive selection are out of line with the PAML results. The extent of agreement between the two estimations (defined by the number of cases where PAML and substitution mapping agree on whether a dN/dS value is above or below 1, divided by the total number of estimations) is 0.92. PAML detects more positive selection events (3,029 versus 384 for mapping), but 88% of them are due to unreliable dN/dS values ranging from 3 to more than 1000. The substitution mapping is thus more stringent than PAML and seems to restrict the number of potential false detections of selection events, a key feature to help separate the wheat from the chaff in future studies of datasets with thousands of species.

Providing more reliable estimations, BppML+MapNH substitution mapping is able to perform a 1 month PAML task in less than half a-day. Fast, robust and stringent, substitution mapping appears to be tailored for dealing with future large dataset analyses.

### Conclusion

Both simulations and real case studies clearly indicate that substitution mapping only requires a simple homogeneous model for branches and a phylogenetic tree with approximate branch lengths to achieve results similar to those generated by complex, non-homogeneous models.

Substitution mapping, which is more robust, not prone to over-parametrization problems and much faster (up to more than 25 000 times compared to non-homogeneous models), appears to be a good descriptor of molecular evolution. However, contrary to non-homogeneous models, it does not provide a framework for hypothesis testing though e.g. likelihood ratio tests.

Thanks to high throughput sequencing, biology has reached a new stage where commonly-used methods push computers to their limits. To overcome these limits, more efficient tools [16] are needed. Moreover, as large amounts of data go together with automated analysis pipelines, model misspecification and high false discovery rate become prominent concerns. In this respect, the application of robust statistics is particularly important. For these reasons, we consider that substitution mapping is one promising avenue for studies in molecular evolution, and could facilitate complex analyses such as the detection of natural selection in huge datasets that could not be dealt with using parameter-rich heterogeneous models.

## Materials and Methods

### Simulation

Simulations were performed with programs developed in C++ using the Bio++ libraries [43]. Initial branch lengths of the tree (same topology than [23]) were obtained from real data, i.e. 987 orthologous genes from 33 mammals obtained from the OrthoMam database [29]. We drew branch-specific Omega values from a gamma distribution of mean 0.2 (shape 0.5) and simulated 50 sequence alignments of 1000 sites each.

Third positions of the same orthologous genes were used for nucleotide simulations (GC analysis). Theta values (one per branch) were drawn from a uniform distribution of between 0 and 1.

### Substitution mapping

The procedure is an extension of that described by Dutheil et al [1], which only provides the mean number of substitutions per branch and per site in the alignment. Here, we also estimated the detail counts for each type of substitution (e.g. synonymous or non-synonymous, AT→GC or GC→AT).

At each position *i* in the alignment, we computed the substitution vector 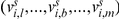 where 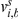 is the posterior estimate of the number of substitutions of type *s* that occurred on branch *b* for *m* branches in the tree. 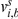 was estimated by averaging all possible ancestral states at top 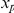 and bottom 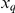 nodes [1]:
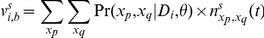



Given the data and parameters, is the joint probability of having state 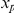 at the bottom node and state 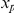 at the top node. It is computed as follows [1], [44], [45]:
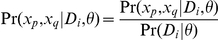



The denominator is the likelihood for site *i* [46], while the numerator is obtained in a very similar way, but considering the ancestral states 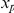 and 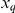 as known in the Felsenstein recursion. Given the initial state 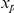 and final state 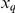, term 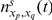 is the mean number of substitutions of type s that occurs on a branch of length t. To compute this mean number, we used the uniformization method [14], [15] as it is exact, numerically more stable than the method proposed in Dutheil et al [1], and provides counts for each type of substitution.

Substitution counts obtained for each site were summed, then pooled depending on the type of data: AT→GC and GC→AT for nucleotides, and synonymous vs non-synonymous for codon sequences. On amino-acid sequences, we could have pooled substitutions in conservative vs non-conservative substitutions.

Substitution mapping was implemented in the MapNH program built using Bio++ libraries (Dutheil et al., 2006). The uniformization method for counting substitution was implemented from the R code kindly provided by Tataru and Hobolth [15], and made available to the libraries (version 2.0.2 and later). MapNH is available in the TestNH package at http://biopp.univ-montp2.fr/forge/testnh, as source code and binary versions. The program is written in standard C++ and it compiles in Linux, Windows and MacOS systems, and depends only on Bio++ libraries.

### Mitochondrial genomes of mammals

Mitochondrial genomes for the 139 mammals were obtained from NCBI. Body mass values were obtained from the AnAge database [47].

### Statistics

Statistical analyses were performed and graphs drawn up with the R software [48].

We used median absolute deviation (defined as 
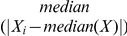
) instead of 95% intervals to measure dispersion in Figure 1 and 2. 95% intervals were highly dominated by outliers, and the resulting measure of dispersion reflected false distinction between model performances.

## Supporting Information

Figure S1 **Tree used in simulations.** Initial branch lengths of the tree (same topology than [23]) were obtained from real data, i.e. 987 orthologous genes from 33 mammals obtained in the OrthoMam database [29].
(TIF)

Figure S2 **Estimated GC equilibrium approximation under various substitution mapping conditions compared with real GC equilibrium approximation from simulations.** GC equilibrium approximation is defined by, where AT→GC and GC→AT are number of substitutions of each type inferred by substitution mapping. Each point represents the median of one of the five classes, confidence intervals are the median absolute deviation of their corresponding class. The real distribution is plotted to give an idea of the initial variability in the values to estimate.
(TIF)

Figure S3 **Relative error in GC equilibrium estimation by branch and by site.** Panel A is the relative error variation in GC equilibrium approximation of a branch according to its length. Each point represents the median of one of the six classes, bars represent the median absolute deviation of its corresponding class. Panel B is relative error in GC equilibrium approximation of a site according to its AT to GC substitution number (top plot), and GC to AT substitution number (bottom plot). Each point represents the median of one of the 17 classes, bars represent the median absolute deviation of its corresponding class. The top axis gives the number of sites concerned by the corresponding number of substitutions.
(TIF)

## References

[1] Dutheil J, Pupko T, Jean-Marie A, Galtier N (2005). A model-based approach for detecting coevolving positions in a molecule.. Molecular biology and evolution.

[2] Dimmic MW, Hubisz MJ, Bustamante CD, Nielsen R (2005). Detecting coevolving amino acid sites using Bayesian mutational mapping.. Bioinformatics.

[3] Dutheil J (2008). Detecting site-specific biochemical constraints through substitution mapping.. Journal of molecular evolution.

[4] Mayrose I, Otto SP (2011). A likelihood method for detecting trait-dependent shifts in the rate of molecular evolution.. Molecular biology and evolution.

[5] Zhai W, Slatkin M, Nielsen R (2007). Exploring Variation in the dN/dS Ratio Among Sites and Lineages Using Mutational Mappings: Applications to the Influenza Virus.. Journal of Molecular Evolution.

[6] Lartillot N (2006). Conjugate Gibbs sampling for Bayesian phylogenetic models.. Journal of computational biology a journal of computational molecular cell biology.

[7] Robinson DM, Jones DT, Kishino H, Goldman N, Thorne JL (2003). Protein evolution with dependence among codons due to tertiary structure.. Molecular Biology and Evolution.

[8] Shindyalov IN, Kolchanov NA, Sander C (1994). Can three-dimensional contacts in protein structures be predicted by analysis of correlated mutations?. Protein Engineering.

[9] Nielsen R (2002). Mapping mutations on phylogenies.. Systematic biology.

[10] Dutheil J, Galtier N (2007). Detecting groups of coevolving positions in a molecule: a clustering approach.. BMC Evolutionary Biology.

[11] Felsenstein J (2004). Inferring Phylogenies..

[12] Yang Z (2006). Computational molecular evolution..

[13] Rodrigue N, Philippe H, Lartillot N (2008). Uniformization for sampling realizations of Markov processes: applications to Bayesian implementations of codon substitution models.. Bioinformatics.

[14] Hobolth A, Stone EA (2009). Simulation from endpoint-conditioned, continuous-time Markov chains on a finite state space, with applications to molecular evolution.. The Annals of Applied Statistics.

[15] Tataru P, Hobolth A (2011). Comparison of methods for calculating conditional expectations of sufficient statistics for continuous time Markov chains.. BMC Bioinformatics.

[16] Minin VN, Suchard Ma (2008). Fast, accurate and simulation-free stochastic mapping.. Philosophical transactions of the Royal Society of London Series B, Biological sciences.

[17] Hobolth A, Jensen J (2005). Applications of hidden Markov models for comparative gene structure prediction.. J Comput Biology.

[18] Jobson RW, Nabholz B, Galtier N (2010). An evolutionary genome scan for longevity-related natural selection in mammals.. Molecular Biology and Evolution.

[19] Duret L, Arndt PF (2008). The impact of recombination on nucleotide substitutions in the human genome.. PLoS genetics.

[20] Paland S, Lynch M (2006). Transitions to asexuality result in excess amino acid substitutions.. Science (New York, N Y).

[21] Popadin K, Polishchuk LV, Mamirova L, Knorre D, Gunbin K (2007). Accumulation of slightly deleterious mutations in mitochondrial protein-coding genes of large versus small mammals.. Proceedings of the National Academy of Sciences of the United States of America.

[22] Boussau B, Blanquart S, Necsulea A, Lartillot N, Gouy M (2008). Parallel adaptations to high temperatures in the Archaean eon.. Nature.

[23] Romiguier J, Ranwez V, Douzery EJP, Galtier N (2010). Contrasting GC-content dynamics across 33 mammalian genomes: Relationship with life-history traits and chromosome sizes.. Genome research.

[24] Galtier N, Gouy M (1998). Inferring pattern and process: maximum-likelihood implementation of a nonhomogeneous model of DNA sequence evolution for phylogenetic analysis.. Molecular biology and evolution.

[25] Nielsen R, Yang Z (1998). Likelihood models for detecting positively selected amino acid sites and applications to the HIV-1 envelope gene.. Genetics.

[26] Lartillot N, Poujol R (2011). A phylogenetic model for investigating correlated evolution of substitution rates and continuous phenotypic characters.. Molecular Biology and Evolution.

[27] Yang Z (1998). Likelihood ratio tests for detecting positive selection and application to primate lysozyme evolution.. Molecular biology and evolution.

[28] Proux E, Studer RA, Moretti S, Robinson-Rechavi M (2009). Selectome: a database of positive selection.. Nucleic Acids Research.

[29] Ranwez V, Delsuc F, Ranwez S, Belkhir K, Tilak M-K (2007). {OrthoMaM:} A database of orthologous genomic markers for placental mammal phylogenetics.. {BMC} Evolutionary Biology.

[30] Jukes TH, Cantor CR, Munro HN (1969). Evolution of protein molecules.. Mammalian Protein Metabolism.

[31] Tamura K (1992). Estimation of the number of nucleotide substitutions when there are strong transition-transversion and G+C content biases.. Molecular Biology and Evolution.

[32] Pollard KS, Salama SR, Lambert N, Lambot M-A, Coppens S (2006). An RNA gene expressed during cortical development evolved rapidly in humans.. Nature.

[33] Ohta T (1973). Slightly deleterious mutant substitutions in evolution.. Nature.

[34] Barraclough TG, Fontaneto D, Ricci C, Herniou EA (2007). Evidence for inefficient selection against deleterious mutations in cytochrome oxidase I of asexual bdelloid rotifers.. Molecular Biology and Evolution.

[35] Neiman M, Hehman G, Miller JT, Logsdon JM, Taylor DR (2010). Accelerated mutation accumulation in asexual lineages of a freshwater snail.. Molecular biology and evolution.

[36] Pond SLK, Frost SDW, Muse SV (2005). HyPhy: hypothesis testing using phylogenies.. Bioinformatics.

[37] Dutheil J, Boussau B (2008). Non-homogeneous models of sequence evolution in the Bio++ suite of libraries and programs.. BMC Evolutionary Biology.

[38] Escobar JS, Glémin S, Galtier N (2011). GC-Biased Gene Conversion Impacts Ribosomal DNA Evolution in Vertebrates, Angiosperms and Other Eukaryotes.. Molecular Biology.

[39] Duret L, Galtier N (2009). Biased Gene Conversion and the Evolution of Mammalian Genomic Landscapes.. Annual Review of Genomics and Human Genetics.

[40] Nickel GC, Tefft D, Adams MD (2008). Human PAML browser: a database of positive selection on human genes using phylogenetic methods.. Nucleic acids research.

[41] Liberles D, Schreiber D, Govindarajan S, Chamberlin S, Benner S (2001). The Adaptive Evolution Database (TAED).. Genome Biology.

[42] Genome 10K Community of Scientists (2009). Genome 10K: a proposal to obtain whole-genome sequence for 10,000 vertebrate species.. The Journal of heredity.

[43] Dutheil J, Gaillard S, Bazin E, Glémin S, Ranwez V (2006). Bio++: a set of C++ libraries for sequence analysis, phylogenetics, molecular evolution and population genetics.. BMC Bioinformatics.

[44] Pupko T, Sharan R, Hasegawa M, Shamir R, Graur D (2003). Detecting excess radical replacements in phylogenetic trees.. Gene.

[45] Galtier N, Boursot P (2000). A new method for locating changes in a tree reveals distinct nucleotide polymorphism vs divergence patterns in mouse mitochondrial control region.. Journal of Molecular Evolution.

[46] Felsenstein J (1981). Evolutionary trees from DNA sequences: a maximum likelihood approach.. Journal of Molecular Evolution.

[47] De Magalhaes J, Costa J (2009). A database of vertebrate longevity records and their relation to other life-history traits.. Journal of Evolutionary Biology.

[48] Team RDC (2008). R: A Language and Environment for Statistical Computing.. R Foundation for Statistical Computing.

