# Supplementary figures and images for: Fast and Robust Characterization of Time-Heterogeneous Sequence Evolutionary Processes Using Substitution Mapping
Source: PLoS One. 2012 Mar 27;7(3):e33852. doi: 10.1371/journal.pone.0033852 (PMC3313935; doi:10.1371/journal.pone.0033852)

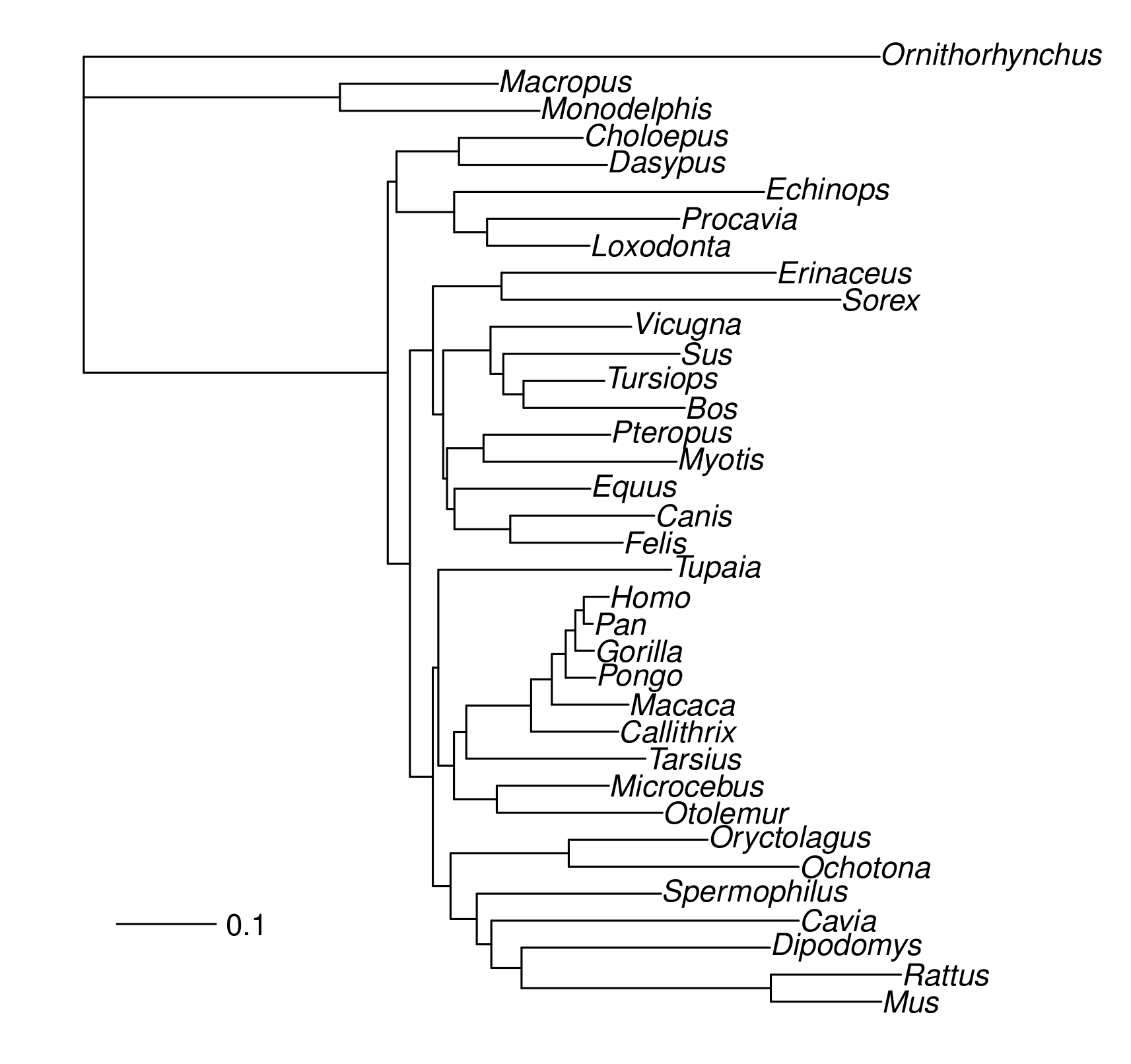

Supplement: Figure S1 — Tree used in simulations. Initial branch lengths of the tree (same topology than [23]) were obtained from real data, i.e. 987 orthologous genes from 33 mammals obtained in the OrthoMam database [29]. (TIF) [file pone.0033852.s001.tif]

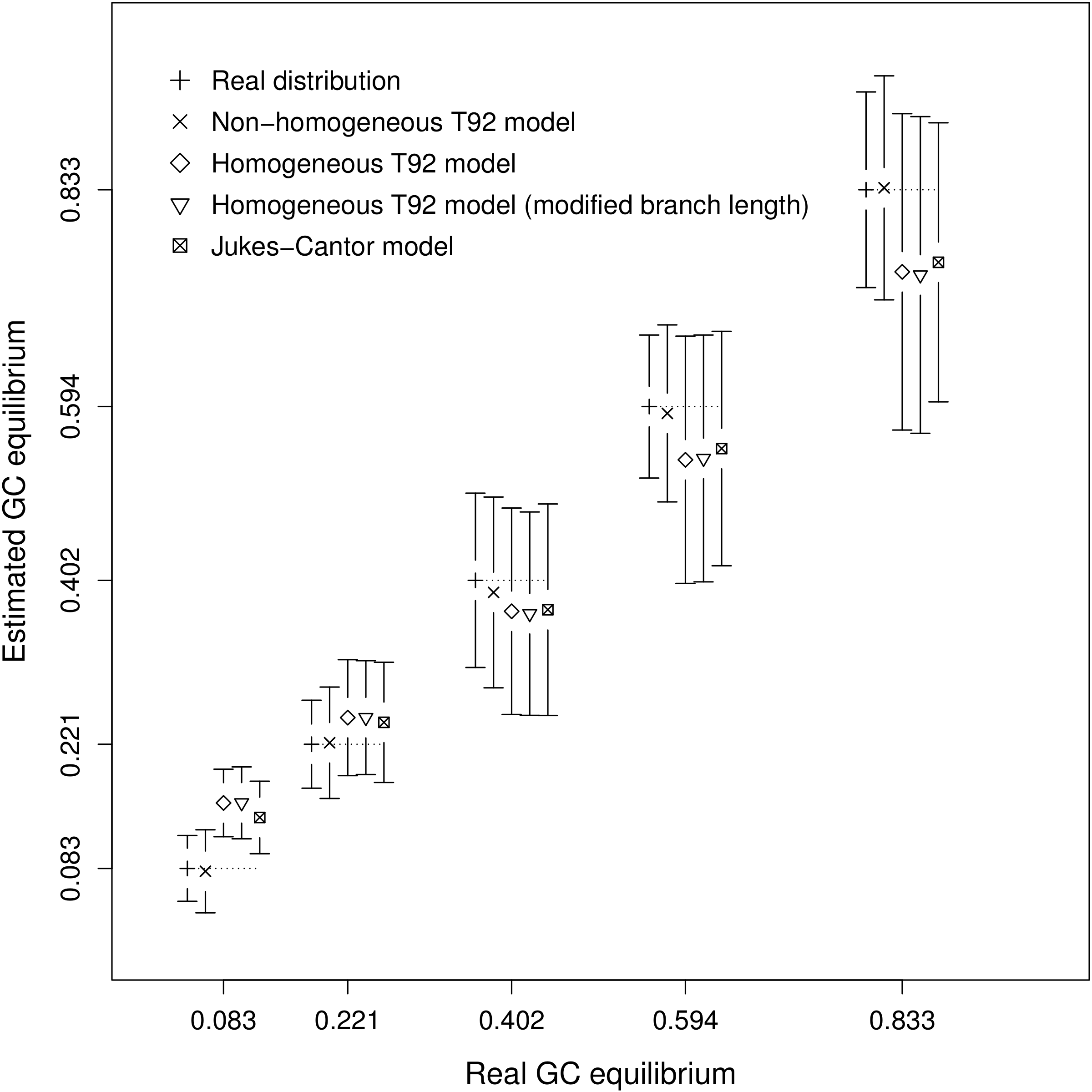

Supplement: Figure S2 — Estimated GC equilibrium approximation under various substitution mapping conditions compared with real GC equilibrium approximation from simulations. GC equilibrium approximation is defined by, where AT→GC and GC→AT are number of substitutions of each type inferred by substitution mapping. Each point represents the median of one of the five classes, confidence intervals are the median absolute deviation of their corresponding class. The real distribution is plotted to give an idea of the initial variability in the values to estimate. (TIF) [file pone.0033852.s002.tif]

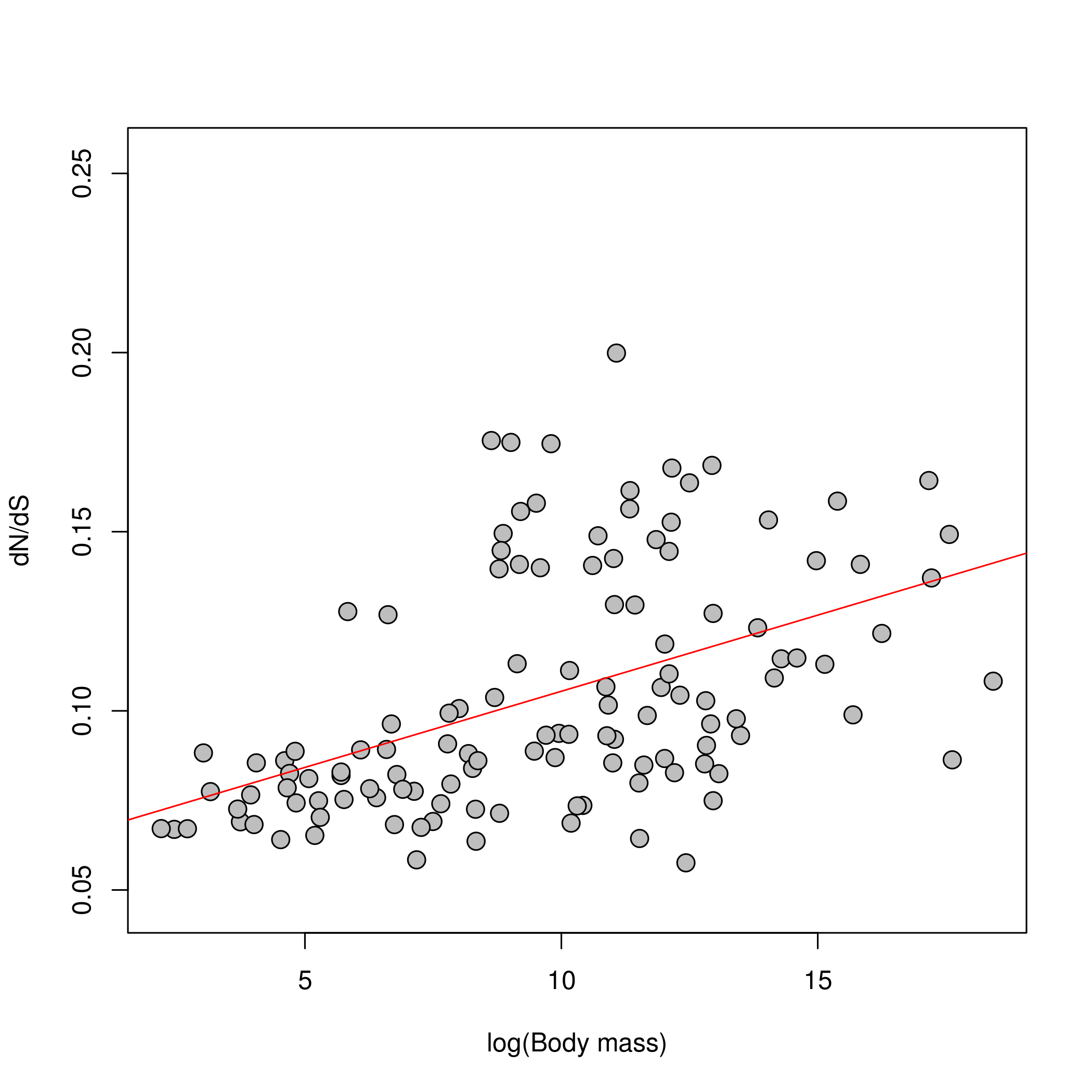

Supplement: Figure S3 — Relative error in GC equilibrium estimation by branch and by site. Panel A is the relative error variation in GC equilibrium approximation of a branch according to its length. Each point represents the median of one of the six classes, bars represent the median absolute deviation of its corresponding class. Panel B is relative error in GC equilibrium approximation of a site according to its AT to GC substitution number (top plot), and GC to AT substitution number (bottom plot). Each point represents the median of one of the 17 classes, bars represent the median absolute deviation of its corresponding class. The top axis gives the number of sites concerned by the corresponding number of substitutions. (TIF) [file pone.0033852.s003.tif]
